# 5′ Untranslated mRNA Regions Allow Bypass of Host Cell Translation Inhibition by Legionella pneumophila

**DOI:** 10.1128/iai.00179-22

**Published:** 2022-11-02

**Authors:** Erion Lipo, Seblewongel Asrat, Wenwen Huo, Asaf Sol, Christopher S. Fraser, Ralph R. Isberg

**Affiliations:** a Program in Genetics, Tufts University School of Medicine, Boston, Massachusetts, USA; b Program in Molecular Microbiology, Tufts University School of Medicine, Boston, Massachusetts, USA; c Department of Molecular and Cellular Biology, University of California, Davis, California, USA; d Department of Molecular Biology and Microbiology, Tufts University School of Medicine, Boston, Massachusetts, USA; Yale University School of Medicine

**Keywords:** *Legionella pneumophila*, innate immunity, interferon, macrophages, translation inhibition, translation initiation

## Abstract

Legionella pneumophila grows within membrane-bound vacuoles in alveolar macrophages during human disease. Pathogen manipulation of the host cell is driven by bacterial proteins translocated through a type IV secretion system (T4SS). Although host protein synthesis during infection is arrested by the action of several of these translocated effectors, translation of a subset of host proteins predicted to restrict the pathogen is maintained. To identify the spectrum of host proteins selectively synthesized after L. pneumophila challenge, macrophages infected with the pathogen were allowed to incorporate the amino acid analog azidohomoalanine (AHA) during a 2-h time window, and newly synthesized macrophage proteins were isolated by orthogonal chemistry followed by mass spectrometry. Among the proteins isolated were interferon-stimulated genes as well as proteins translated from highly abundant transcripts. Surprisingly, a large number of the identified proteins were from low-abundance transcripts. These proteins were predicted to be among the most efficiently translated per unit transcript in the cell based on ribosome profiling data sets. To determine if high ribosome loading was a consequence of efficient translation initiation, the 5′ untranslated regions (5′ UTR) of transcripts having the highest and lowest predicted loading levels were inserted upstream of a reporter, and translation efficiency was determined in response to L. pneumophila challenge. The efficiency of reporter expression largely correlated with predicted ribosome loading and lack of secondary structure. Therefore, determinants in the 5′ UTR allow selected host cell transcripts to overcome a pathogen-driven translation blockade.

## INTRODUCTION

Diseases caused by intracellular pathogens remain significant and persistent global public health problems. Many of these organisms, such as Mycobacterium tuberculosis and the sexually transmitted Chlamydia trachomatis, grow intravacuolarly in membrane-bound compartments within host cells (1, 2). Legionella pneumophila is one such Gram-negative intracellular pathogen that proliferates within host cells (3). The organism was initially identified as the causative agent of Legionnaires’ disease, resulting from inhaled aerosols of water sources contaminated with amoebae bearing replicating L. pneumophila (4–6). Once inhaled by the susceptible host, *Legionella* transfers its site of replication from amoebae to alveolar macrophages, with the potential for causing life-threatening atypical pneumonia (7, 8), particularly in immunocompromised individuals and the elderly (8).

The replication vacuole that bears *Legionella* avoids trafficking to the lysosome as a consequence of proteins translocated through the bacterial type IV secretion system (9–13). Called Icm/Dot, this secretion system translocates over 300 proteins and is absolutely required for creating a replication niche and preventing host restriction (11, 14). The translocated effector proteins promote the recruitment of endoplasmic reticulum-derived vacuolar components and modulate the host anti-pathogen response (15). These include bacterial proteins that control host vesicular trafficking, block host cell translation, and block the evolutionarily conserved host autophagy system (16).

The host can detect intracellular microbes through innate immune sensing by pattern-recognition receptors (PRRs) such as Toll-like receptors (TLR) and Nod-like receptors, which recognize a variety of pathogen-associated molecular patterns, including lipopolysaccharide and peptidoglycan derivatives (17). Upon PRR activation, the innate immune response triggers the activation of transcription factors, such as those regulated by NF-κB, to transcribe proinflammatory cytokines and chemokines (18). PRR activation of inflammasomes combined with effector-triggered response leads to a robust induction and secretion of the interleukin-1 (IL-1) family of cytokines during L. pneumophila infection. Infected cells, however, are poor producers of IL-6, tumor necrosis factor (TNF), and IL-12. The production and secretion of IL-1α and IL-1β by infected cells lead to the activation of uninfected bystander cells which produce IL-6, TNF, and IL-12, which are important for bacterial clearance (19).

A common strategy used by intracellular pathogens is to inactivate components of the host protein synthesis machinery to undermine host restriction (10, 20–23). Diphtheria toxin, shiga toxin, and Pseudomonas exotoxin A are examples of proteins that directly modulate host translation (24–26). Although there are diverse explanations for the conservation of this tactic, it likely allows immune evasion when pathogens encounter hosts. L. pneumophila translocates at least seven proteins that depress host cell translation efficiency. Three of these proteins (Lgt1, Lgt2, Lgt3) are glucosyltransferases that modify and inactivate the host translation elongation factor 1A (eEF1a) (21). As a consequence of these secreted effector activities, a panel of host cell transcripts is induced, which has been dubbed the effector-triggered response (22). This response involves the induction of both NF-κB as well as mitogen-activated protein kinase (MAPK)-dependent transcripts (27–32). There also appears to be a host-driven response that inhibits translation initiation after *Legionella* infection, which may have effects on transcriptional induction (33).

The inhibition of translation, which drives increased host cell transcription, has the paradoxical consequence that pathogen-response transcripts are predicted to be poorly translated due to elongation inhibition (29). In spite of the low translation efficiency, proinflammatory cytokines such as tumor necrosis factor (TNF) and the interleukins IL-1α and IL-1β are clearly produced within cells harboring the bacterium (20, 29). Arguing for the importance of this response in restricting bacterial growth, mice that are defective for IL-1α and IL-1β production and anti-TNF-α-treated rheumatoid arthritis patients are at high risk for L. pneumophila infection (20, 34). These results are consistent with the model that the host is able to mount an immune response in the face of translation inhibition. Alternatively, the critical cytokine response necessary to clear the organism could be provided by uninfected bystander cells during disease, without the involvement of cytokines from infected cells, as has been shown in the mouse pneumonia model (35).

The ability of infected cells to produce inflammatory cytokines while the pathogen inhibits protein synthesis indicates that there must be mechanisms to allow translation in the face of these antagonists (29). The primary accepted explanation for the selective translation of inflammatory cytokines in infected cells is that the proteins synthesized in response to L. pneumophila originate from the most abundantly expressed transcripts in these cells (36). This model argues that the synthesis of proinflammatory cytokines and chemokines occurs in infected cells as a consequence of 1,000× transcriptional induction in response to L. pneumophila, allowing selective protein synthesis in the face of elongation inhibition (36). Here, we test this model by identifying proteins synthesized within the infected subpopulation of cells during a 2-h time period commencing at 4 h postinfection. We show that in addition to proteins derived from highly transcribed genes, a large subset originates from genes having preferential ribosome loading without corresponding largescale transcription. As a consequence, efficient translation initiation bypasses the blockade caused by pathogen attacks.

## RESULTS

### Identification of host proteins that are selectively translated in the presence of *Legionella* infection.

**FIG 1:**
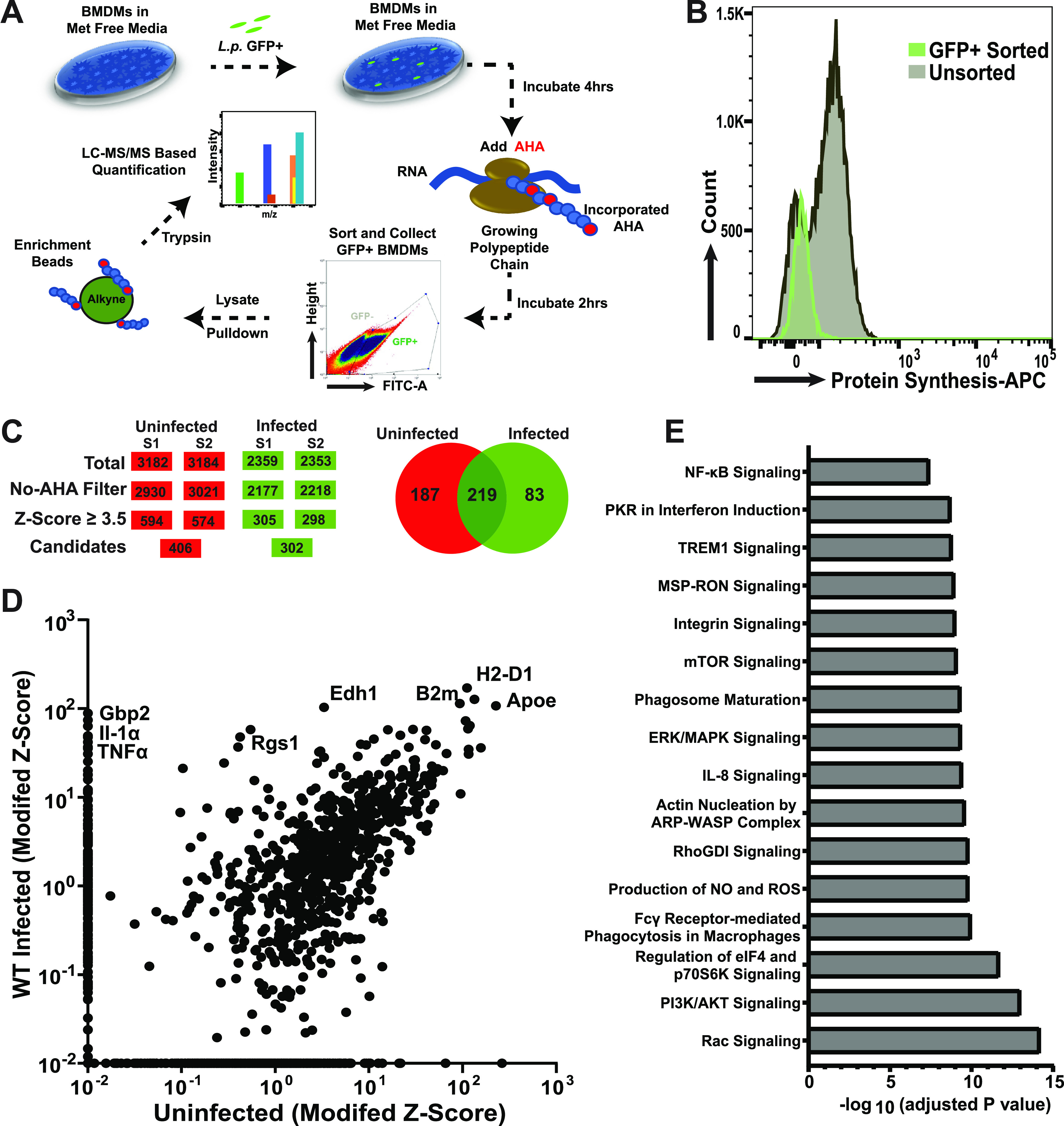
AHA labeling of BMDMs identifies 302 proteins selectively synthesized after L. pneumophila challenge. (A) Strategy for identification of newly synthesized proteins in macrophages infected with L. pneumophila. Methionine-starved C57BL/6 BMDMs were challenged with L. pneumophila*-*GFP at a multiplicity of infection (MOI) of 15 and incubated for 4 h. AHA was introduced into the medium, and infection was allowed to proceed for an additional 2 h. Cells were then harvested and subjected to cell sorting, gating on GFP expression to identify macrophages with associated bacteria. The sorted cells from the GFP^+^ gate were lysed and incubated with alkyne-coated beads in the presence of Cu^2+^ to allow covalent linkage of AHA-incorporated nascent chains to resin (see Materials and Methods). An uninfected sample was also run in parallel. The covalently linked proteins with AHA incorporated were released from the resin by trypsin digestion and then subjected to LC-MS/MS analysis to identify all proteins translated during this time frame. (B) BMDMs challenged with L. pneumophila were blocked for protein synthesis. Shown is flow cytometry analysis of BMDMs cells infected with L. pneumophila at MOI = 15 for 6 h allowing for 2 h AHA incorporation as in panel A. Sorted cells were fixed, permeabilized, and incubated with APC-phosphine to allow detection of AHA incorporation. (C) Flow chart demonstrating strategy for identification of proteins by mass spectrometry (MS). Modified Z-score analysis was performed (see Materials and Methods) (73). To determine nonspecific binding to the alkyne resin, a control sample was used with the omission of AHA. (D) Modified Z-scores for protein candidates identified by MS in uninfected and infected BMDMs. Proteins that were absent from a sample were given a value of 0.001 as the lowest limit of detection (Data Set S1). (E) IPA-bases enriched pathway analysis of the list of newly synthesized proteins A (Qiagen Inc; https://digitalinsights.qiagen.com) Proteins identified, values, and statistical significance are described in Data Set S1.

We identified the proteins encoded by mouse bone marrow-derived macrophages (BMDMs) that were selectively translated in response to L. pneumophila-green fluorescent protein-positive infection during a 2-h window. To this end, a snapshot proteomics strategy was performed, in which the methionine analog AHA was added from 4 to 6 h postbacterial challenge (37). The azido moiety on AHA provides a target for the covalent linkage of newly synthesized proteins to alkyne-modified resin via orthogonal chemistry, permitting subsequent pelleting of resin and analysis (38) (Fig. 1A). After the 2-h incubation, BMDMs were harvested and sorted based on green fluorescent protein (GFP) fluorescence, a proxy for cells harboring L. pneumophila-GFP^+^. The BMDMs were lysed, and the lysate was subjected to orthogonal Cu^2+^ chemistry to allow the linkage of newly synthesized proteins to the alkyne beads before pulldown and identification by liquid chromatography-tandem mass spectrometry (LC-MS/MS) (see Materials and Methods). Before lysing, a portion of these cells was fixed and permeabilized, and AHA-labeled proteins were covalently linked to allophycocyanin (APC)-phosphine to measure translation as a function of fluorescence intensity (Fig. 1B). Infected cells, sorted based on GFP fluorescence, showed a mean APC fluorescence intensity that was 5% the level of the bystander uninfected cells. Therefore, the sorted infected cell population that was subjected to proteomic analysis was strongly depressed for translation, consistent with previous observations on this population (29).

Analysis of proteins proteolytically released from alkyne beads and analyzed by LC-MS/MS was performed on duplicate infections prepared on different days, with the rank-order results displayed as modified Z-scores for each candidate (39). To identify candidates, a relatively stringent Z >3.5 was used to ensure that extreme outliers will be retained as candidates, defined as proteins that show predominance relative to other components of the sample. To control for nonspecific binding to the alkyne resin, samples were used with the omission of AHA, and this group was filtered out from the outliers identified in the AHA-treated sample (Fig. 1C). By comparing infected to naive uninfected BMDMs, we were able to identify 83 proteins that were overrepresented in cells harboring L. pneumophila (Fig. 1D and E; Data Set S1).

**FIG 2:**
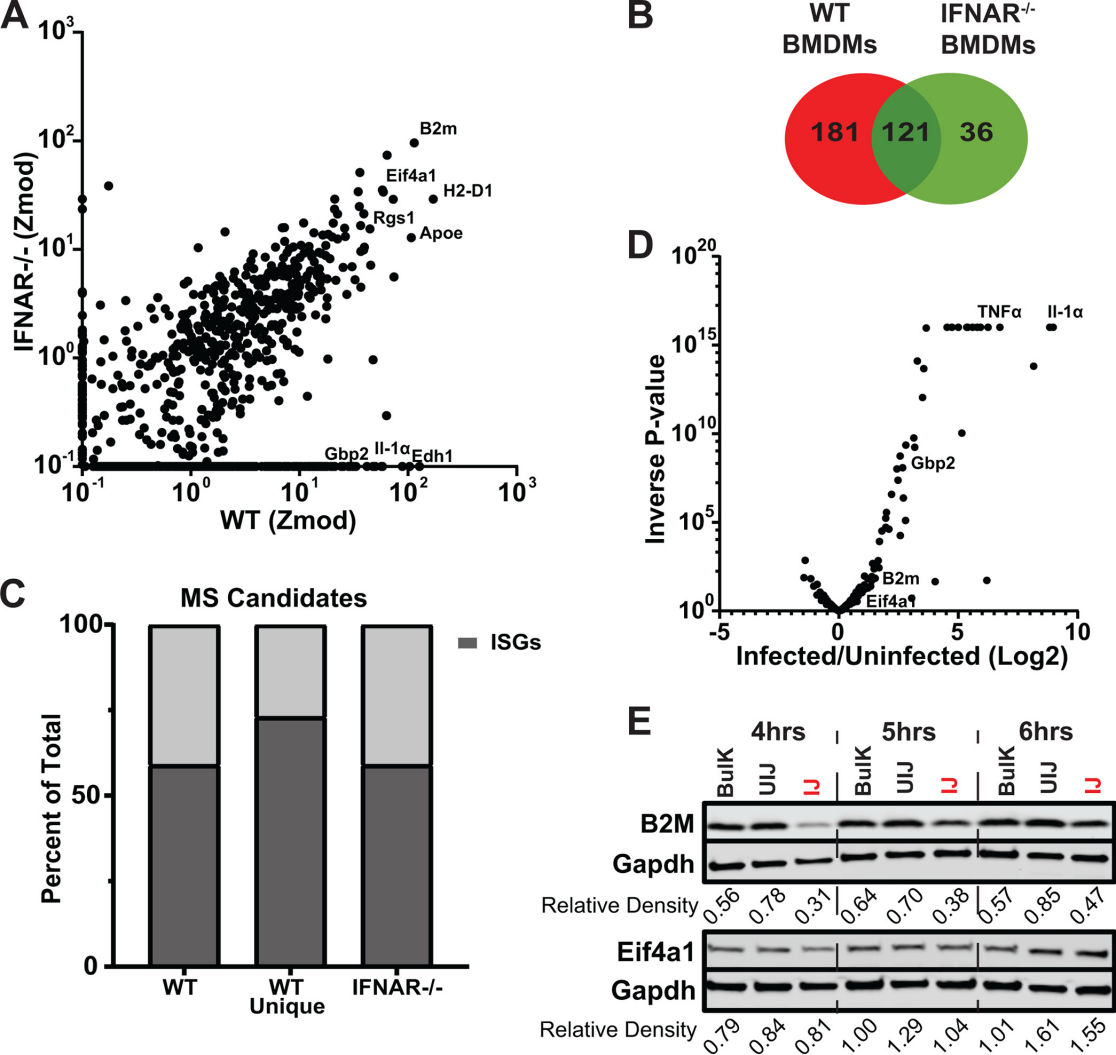
Dependence of identified translated host proteins on IFNa receptor signaling. (A) Modified Z-score analysis plot comparison between WT and IFNAR^−/−^ BMDMs infected with L. pneumophila. (B) Venn diagram comparing candidates with a Z-score ≥3.5 in WT and IFNAR^−/−^ BMDMs challenged with L. pneumophila. (C) The ISG signatures of WT (total hits and hits unique to WT) and IFNAR^−/−^ BMDMs candidates as identified by the Interferome Database (see Materials and Methods). (D) Transcriptome analysis of BMDMs data set in the presence and absence of L. pneumophila infection. Shown is fold change in the presence of infection for the candidates identified by MS. 77% of candidates cluster (*P* > 0.05, Wald test) with no evidence of transcriptional induction. Data for Fig. 2A to D are detailed in Data Set S2. (E) BMDMs were infected with L. pneumophila-GFP (Δ*flaA)* at MOI = 15 and sorted 6 h postinfection based on GFP intensity (experiment was performed as two biological replicates; one is shown). Immunoblot analysis of B2M and EIF4a1 shows increased accumulation of proteins over time after challenge with L. pneumophila WT. Bulk, total population before sorting; UI, uninfected (GFP^−^ cells); IJ, infected (GFP^+^) cells. Relative density below blots refers to densitometry in sorted cells of noted proteins normalized to Gapdh.

It should be noted that proteins identified by this method favor high molecular weight proteins and those that have a high representation of methionine residues. Despite this limitation, we were able to identify relatively small chemokines and cytokines in the GFP^+^ sample, such as TNF-α, that were not present in samples from cells not exposed to bacterium. Proteins identified by MS were submitted to various bioinformatics database searches (40–42). This approach led to the determination that a large fraction of the candidates appeared to be interferon (IFN)-inducible proteins. The Interferome database contains IFN-stimulated genes (ISGs) curated from publicly available microarray data sets (43). Roughly 60% of the proteins identified in the infected cells were IFN inducible based on these criteria (Fig. 2C). This result is surprising because there is little or no IFN detectable by Western blot or MS (44); however, a number of reports have shown that there is low-level ISG expression as a consequence of basal IFN preexisting in cultured cells (44–46). Focusing on the set of 83 proteins that was unique to infected BMDMs, 80% were ISGs.

Given that the proteins were synthesized without obvious largescale IFN induction, the relative abundance of ISGs was further interrogated by challenging interferon-α/β receptor knockout (IFNAR^−/−^) BMDMs with L. pneumophila. IFNAR binds to type I interferons and is necessary for the upregulation of a set of ISGs in response to this cytokine. Proteomic analysis of IFNAR^−/−^ macrophages was performed identically to the analysis of C57/BL6 (wild-type [WT]) labeling with AHA between 4 and 6 h postinfection, and the infected subpopulation of BMDMs was compared to that of IFNAR^+/+^ macrophages (Fig. 2A; Data Set S2). Overall, there was a 50% decrease in total proteins identified in IFNAR^−/−^ BMDMs challenged with L. pneumophila compared to the WT (Fig. 2A and B). Surprisingly over 50% of proteins identified were still ISGs.

To understand if transcriptional induction of host genes upon L. pneumophila infection explained the selective translation of these ISGs, RNA-sequencing data (36) in WT BMDMs were utilized to determine transcriptional differences among our candidates. Although a fraction of the candidates were transcriptionally upregulated, the majority (77%), however, show no statistically significant change in response to infection of BMDMs (Fig. 2D). To verify that transcripts showing no increase in response to infection were able to continue translation in the presence of L. pneumophila, BMDMs were challenged with L. pneumophila*-*GFP^+^ and separated into infected (IJ) and bystander populations (UIJ). These samples were then probed at 4, 5, and 6 h postinfection with an antibody directed against B2M and EIF4a1 (Fig. 2E), two proteins identified by MS to be encoded by transcripts that show no apparent induction in response to L. pneumophila challenge (Fig. 2D). From the 4- to 6-h time period, there was increased accumulation of both of these proteins in the cells harboring bacteria (IJ), indicating that these proteins were synthesized without corresponding transcriptional activation (Fig. 2E; fraction IJ). Based on the result that a large subset of proteins originated from transcripts that were not transcriptionally induced, we next determined if those transcripts were simply expressed constitutively at high levels, or if a class exists that were translated from low abundance transcripts.

### One class of translated proteins is derived from abundant transcripts.

Previous work noted that the detection of immune response-related proteins in L. pneumophila*-*challenged BMDMs requires the presence of the pattern recognition receptor adaptor protein MyD88 (29, 47, 48). Particularly striking was the fact that challenge of MyD88^−/−^ BMDMs results in high, although clearly attenuated, transcription of genes such as *Il1α* (29). The dependence on MyD88 activity for the detection of these proteins can be explained, as its absence abrogates the superinduction of transcripts, resulting in a correspondingly lower abundance of cytokine and immune response-related proteins (29, 36, 47, 49). Although challenge of MyD88^−/−^ with L. pneumophila reduces the superinduction of transcripts associated with NF-κB-regulated genes, it also allowed us an opportunity to investigate if there exists a class of proteins that are expressed in the presence of L. pneumophila without attendant superinduction.

**FIG 3:**
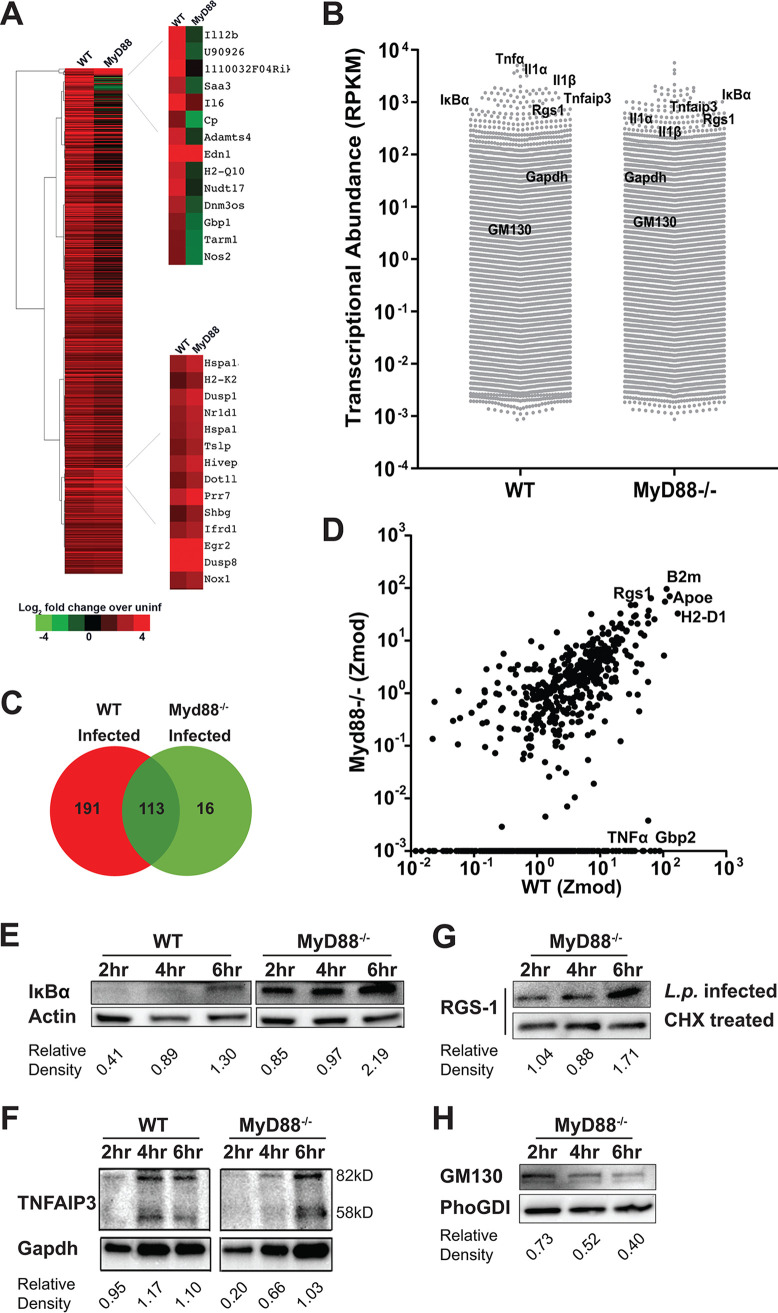
A subset of proteins encoded by highly abundant transcripts was translated in the presence of L. pneumophila infection. (A) Differential transcription in WT and MyD88^−/−^ macrophages infected with L. pneumophila (see Materials and Methods). Data are expressed as fold induction relative to samples of identical mouse macrophage strains incubated in the absence of bacteria. Left: Pearson hierarchical cluster analysis of 800 genes in WT and MyD88^−/−^ macrophages is displayed, showing genes with 2.5-fold change relative to control. Right: clusters showing either differential expression in WT versus MyD88^−/−^ cells or genes similarly upregulated in response to infection in both cell types. (B) Absolute transcript abundance determined from reads per kilobase of exon per million mapped reads (RPKM values) in macrophages harboring bacteria from the C57BL/6 WT (WT) or MyD88^−/−^ harboring macrophages (MyD88). Each data point represents a single gene and a total of 22,006 genes were shown in each group. Genes are noted that show either high expression (in the top 5% of expressed genes) in WT-infected BMDMs, with expression reduced 2 to 10-fold in Myd88^−/−^ BMDMs (TNF-α, IL1α, IL1β, TNFAIP3, IκBα). In addition, genes with similar expression in both groups are noted (GM130, GAPDH). (C and D) Modified Z-score analysis plot comparison between WT and Myd88^−/−^ BMDMs infected with L. pneumophila. Protein candidates were identified after infection with L. pneumophila with a Z-score ≥3.5 in WT and Myd88^−/−^ BMDMs. Venn diagram shows all candidates, not limited to those differentially transcribed (C). Panels A to D are from Data Set S3. (E to H) WT or Myd88^−/−^ BMDMs were challenged with L. pneumophila-GFP (Δ*flaA*) at MOI = 15 and sorted based on GFP^+^ 6 h postinfection with flow cytometry. Immunoblot analysis of (E) IκBα and (F) TNFAIP3 in WT and MyD88^−/−^-infected and sorted cells (experiment was performed as two biological replicates; one is shown). Values show relative density in sorted cells normalized to actin and Gapdh genes, respectively. Immunoblot of RGS-1 (G) and GM130 (H) protein levels in MyD88^−/−^ macrophages challenged with L. pneumophila and sorted 2, 4, and 6 h after challenge (top lane). In parallel, cells were treated with 1 μg/mL cycloheximide (CHX) (G) (bottom lane).

BMDMs from C57BL/6 (WT) and congenic MyD88^−/−^ macrophages were challenged with L. pneumophila and 6 h after infection, RNA was isolated and subjected to transcript analysis by mRNA-seq (see Materials and Methods). Consistent with what has previously been reported (14, 50), challenge of WT BMDMs with L. pneumophila significantly induced transcription of genes encoding proinflammatory cytokines (*Tnf*, *Il1α*, *Il6*), chemokines (*Cxcl1*, *Cxcl2*, *Ccl3*), MAPK genes (*Dusp2*, *Dusp 8*), and NF-κB regulators (*IκB*, *Tnfaip3*) in WT macrophages (Fig. 3A and B; Data Set S3). As expected, the overall induction of genes associated with the innate immune response was attenuated after L. pneumophila challenge of BMDMs from MyD88^−/−^ mice (Fig. 3A and B). To support the model that transcript abundance allows bypass of translation inhibition, we measured absolute transcript levels in both WT and MyD88 deficient macrophages after challenge with L. pneumophila by calculating the number of aligned reads per kilobase of exon per million mapped reads (RPKM values) (Fig. 3B). Transcription of proinflammatory cytokine (*Tnf* and *Il1*) and chemokine (*Cxcl2*, *Ccl2*, *Ccl4*) genes, which represented the most abundantly expressed genes in WT macrophages upon challenge with L. pneumophila (Fig. 3B), was reduced 10× to 100× after challenge MyD88^−/−^ BMDMs, although the level of transcription of *Il1α* and *Il1β* remained relatively high compare to the total population (29, 51).

To determine if transcriptional abundance is the sole determinant that drives bypass of the L. pneumophila translation block, Myd88^−/−^ BMDMs were challenged in duplicate with L. pneumophila, and the proteins translated between 4 and 6 h postinfection were identified by AHA incorporation. To identify the most abundant proteins, a rank order modified Z score ≥3.5 was used in the MyD88^−/−^ BMDMs relative to the WT. In total, there were 129 proteins identified in this fashion after Myd88^−/−^ BMDM infection, 90% of which were also found after infection of the WT (Fig. 3C and D). Furthermore, the 10% that did not meet the stringent significance cutoff after infection WT BMDMs could still be identified by MS in this infection, arguing against any unique proteins expressed in MyD88^−/−^ BMDMs. In contrast, about 60% of the proteins identified in the infected WT BMDMs had a modified Z score <3.5 from the MyD88^−/−^ sample. In fact, 25% of the proteins identified in the infected WT BMDMs were completely absent from MyD88^−/−^ sample (Fig. 3D).

To demonstrate that the set of proteins identified after MyD88^−/−^ infection by MS continue to be translated during the course of L. pneumophila challenge, BMDMs were challenged with L. pneumophila*-*GFP^+^ for 2, 4, and 6 h, with cells collected at each time point and sorted for the GFP^+^ infected populations. Extracts from the sorted cells were then immunoprobed with an antibody directed against IκBα (Fig. 3E) and TNFAIP3 (Fig. 3E and F), two proteins associated with negative regulation of the NF-κB response that were abundantly transcribed in WT BMDMs after L. pneumophila challenge. In both WT and MyD88^−/−^ BMDMs, there was continued accumulation and increased steady-state levels of IκBα in spite of the presence of L. pneumophila protein synthesis inhibition (Fig. 3E) with similar results obtained with TNFAIP3 (Fig. 3F). RGS-1, the regulator of G-protein signaling-1 that is encoded by one of the most abundant transcripts in the MyD88^−/−^ BMDMs, showed similar levels of accumulation (Fig. 3G). To determine the amount of RGS-1 detected by immunoblotting that was due to *de novo* protein synthesis as opposed to long-lived species translated before infection, protein levels after infection were normalized to the amount of protein that remained during cycloheximide treatment and plotted over time. Interestingly, significant accumulation occurred from 4 to 6 h postinfection, a time period previously shown to have maximum levels of protein synthesis inhibition (29). In contrast to these examples, GM-130, which is transcribed at a level that is slightly above the median for MyD88^−/−^ BMDMs infected with L. pneumophila, showed no accumulation after infection (Fig. 3H). These results are consistent with the previously proposed model that transcript abundance in at least a subset of proteins is a determinant for bypassing translation inhibition during *Legionella* infection (36). The results are also consistent with the detection of proteins in this subset being dependent on transcription being above a minimum threshold.

### Identification of a set of proteins synthesized during L. pneumophila infection that is translated from poorly transcribed genes.

**FIG 4:**
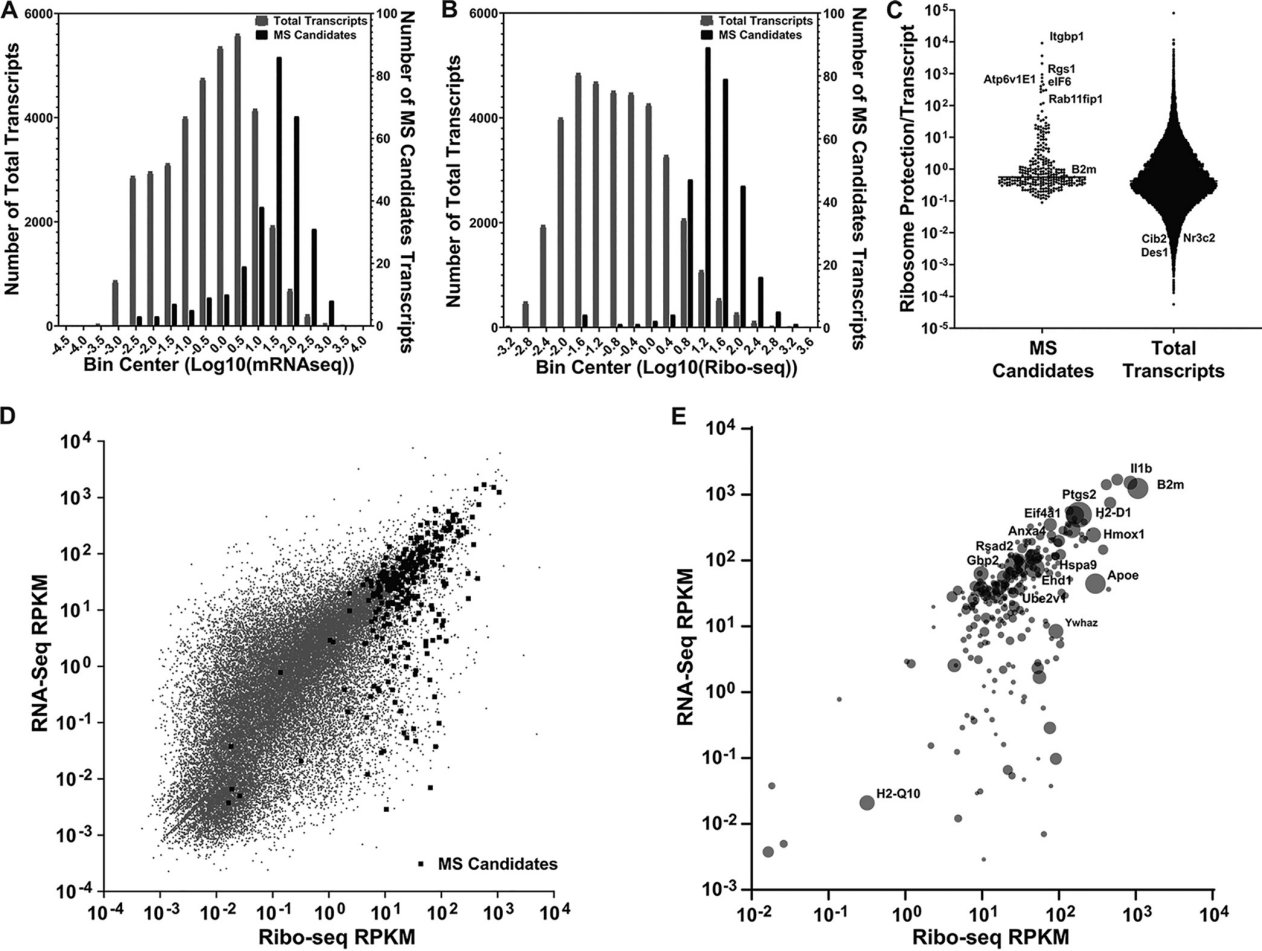
A class of proteins synthesized from low abundance transcripts are translated in the presence of L. pneumophila infection. (A and B) The mRNA-seq (RPKM) and ribosome profiling (RPKM) data are displayed for total transcripts (gray bars) or proteins identified by mass spectrometry (Fig. 1 and 2; black bars). The number of total transcripts or MS candidate transcripts is displayed as a function of read density of transcripts (A) or ribosomal loading (B). Data on *x* axes are placed into 18 bins and based on processed raw data (see Materials and Methods). (C) Translation efficiency (defined as (Ribo-seq RPKM)/(RNA-seq RPKM)) is displayed for each of the MS candidates and total transcripts. (D and E) Ribo-seq and RNA-seq read counts for total transcripts (gray circles) and for proteins identified by mass spectroscopy to be translated in the presence of L. pneumophila infection (black squares). Data from panel D are displayed as relative abundance of proteins identified by mass spectrometry (E). The size of the circle represents the protein abundance in MS. The top 15 most abundant hits have been labeled. RNA-seq and Ribo-seq data are in Data Set S4.

To determine if a class of proteins exists that are synthesized efficiently without originating from abundant transcripts, we analyzed an extensive matched data set that characterizes specific transcript abundancy by both RNA-seq and ribosome profiling after BMDM challenge with L. pneumophila (36). We first compared the entire transcript pool to transcripts encoding the proteins that were identified during the 4 to 6 h postinfection translation snapshot (Fig. 4A; Data Set S4). To analyze these data sets, the log_10_ transcript abundancy for these two populations was placed into bins, and the number of mapped transcripts was plotted as a function of their relative abundancy. There was a striking skewing of the transcript set identified by AHA labeling to the most abundant transcripts (Fig. 4A). In fact, 70% of proteins identified in the translation snapshot were encoded by the most abundant 10% of transcripts. Furthermore, the top 2% of the most abundant transcripts contained 15% of MS-identified candidates. This analysis is consistent with the results arguing that transcript abundance is an important determinant for bypassing L. pneumophila translation inhibition (36).

Despite this general skewing, we still saw a considerable spread in the distribution of transcript abundance, with proteins that were translated from poorly transcribed genes found in the data set. In addition, some proteins from highly abundant transcripts were missing after infection. For instance, IL-1α and TNF-α were not observed in the MyD88^−/−^ samples even though their transcription levels were considerably higher than several proteins identified by MS (Fig. 3B). In contrast, other proteins were encoded by some of the least abundant transcripts in the cell. Therefore, this data set was investigated further to determine if there were other underlying molecular determinants that could explain the identification of proteins encoded by poorly transcribed genes. To this end, the available ribosome profiling (Ribo-Seq) (36) data sets from the same BMDM infections were used to determine if ribosome loading could be a determinant of bypass of L. pneumophila translation inhibition.

Using the identical strategy that we used to analyze transcription abundance, the number of transcripts in the cell was plotted as a function of the ribosome loading for that transcript from the published data set (36), comparing the total pool of transcripts to those encoding the proteins identified in the 4- to 6-h time window (Fig. 4B; Data Set S4). There was a powerful skewing of the proteins that we identified toward the most abundantly loaded transcripts. The top 5% of the most highly loaded transcripts contained 80% of MS-identified candidates. It should be noted, however, that the ribosome loading data are a count of the total amount of transcript protected from nuclease digestion by ribosomes, making the data a function of both the total amount of a particular transcript and the efficiency of loading on that transcript. In fact, when we measured loading efficiency per transcript, by expressing the data as the ratio of Ribo-seq protection/RNA-seq transcript abundance, the MS candidates from the WT infection showed a clear skewing toward heavier protection/transcript. In contrast, the entire population of transcripts showed a near normal distribution of protection (Fig. 4C). Therefore, the MS candidates include proteins encoded by transcripts that show particularly efficient translation relative to the rest of the population.

Based on this analysis, there was a population of proteins identified in our MS data set that appears to be more highly loaded than predicted from transcript abundance. To identify transcripts of low abundance that may be preferentially loaded, we displayed the relative transcript abundance as a function of the total ribosome loading and identified the transcripts that gave rise to the proteins identified after L. pneumophila infection (Fig. 4D). The proteins identified that were encoded by poorly transcribed genes were predominantly skewed toward transcripts that were efficiently loaded. Therefore, these data argue that in addition to transcription abundance, there are sequence determinants that can allow poorly transcribed genes to be translated in the presence of L. pneumophila translation inhibitors.

The relative protein abundance determined by MS was next analyzed to determine if higher transcription levels were connected to yields determined by MS (Fig. 4E). Although MS yields could have been affected by numerous confounding issues, such as the efficiency of AHA incorporation as well as nonuniform fragmentation and peptide flight properties, there was general concordance between the highly expressed transcripts and protein abundance. Notably, proteins with high MS abundance were also identified that were modestly transcribed but heavily ribosome loaded (Fig. 4E). These included the 14-3-3ζ protein (YWHAZ) and apolipoprotein E (apoE), the latter of which was particularly abundant in the MS pool.

### Identification of 5′-UTR sequences that allow efficient translation of low abundance transcripts.

**FIG 5:**
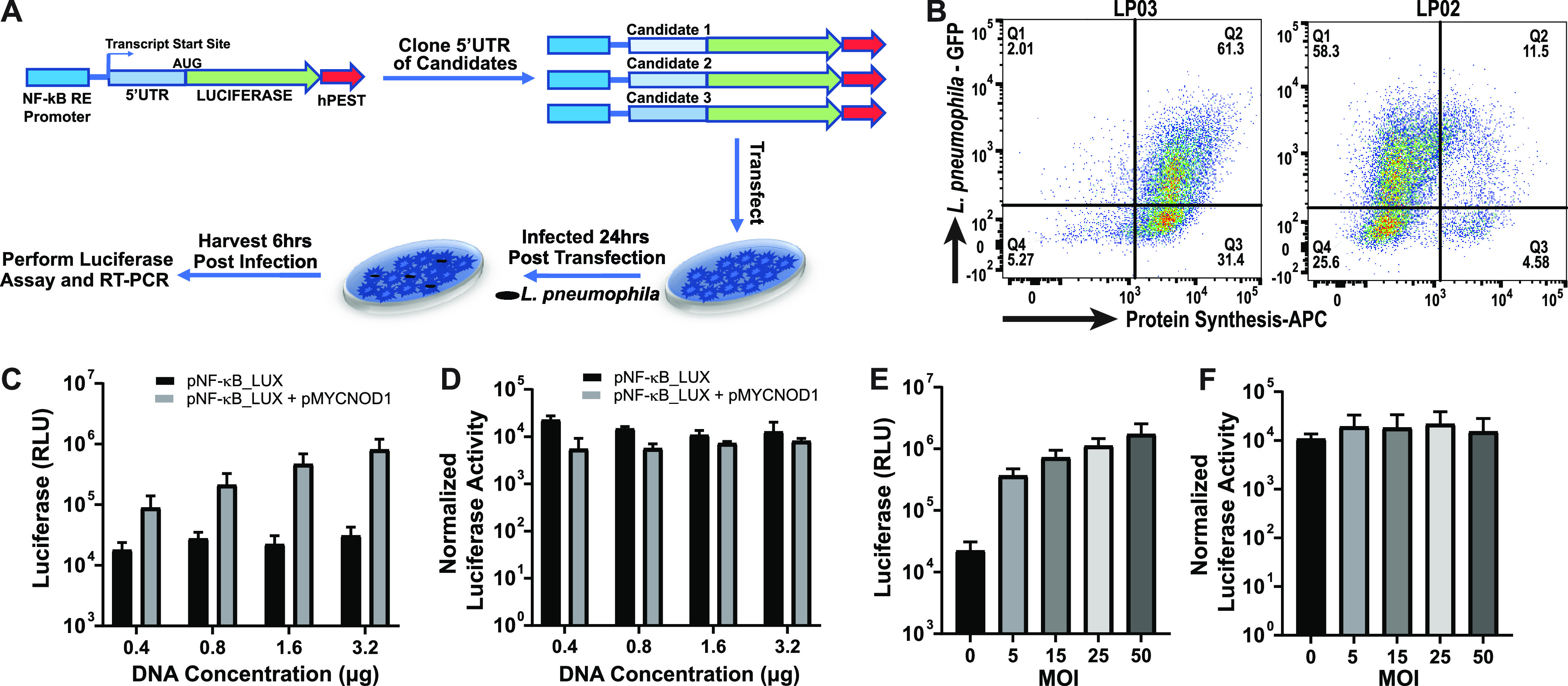
Strategy for determining translation efficiency as a function of 5′-UTR sequence. (A) Experimental approach for evaluating the efficiency of translation driven by 5′-UTR sequences after activation of an NF-κB-responsive promoter in response to L. pneumophila challenge. (B) Flow cytometric analysis of HEK-293T cells challenged with indicated L. pneumophila strains at MOI = 15 for 4 h followed by 2 h further incubation in the presence of AHA. Cells were harvested, fixed, permeabilized, and labeled with APC-phosphine to detect and quantitate protein synthesis (experiment was performed as three biological replicates; one is shown). (C and D) Luciferase activity after transfection with the indicated concentrations of the pNF-kB-LUX plasmid cotransfected with 800 ng of pMYCNOD1 (*n* = 3 biological replicates). (D) RT-PCR was performed to normalize data to the total mRNA level of luciferase and β-actin. (E) Luciferase activity after transfection with the 1.6 μg of pNF-kBLUX plasmid for 24 h and challenged with L. pneumophila at indicated MOI (*n* = 3 biological replicates). (F) RT-PCR was performed to normalize data to the total mRNA level of luciferase and β-actin. RLU, relative luminescence units.

Translation initiation is thought to be the rate-limiting step of protein synthesis, controlled by the efficiency of loading at the 5′ end of transcripts (52). Given that a number of our identified candidates were predicted to be synthesized from heavily ribosome-loaded transcripts, we tested the model that the 5′ UTR contributes to an unexpectedly high abundance of proteins from a subset of poorly transcribed genes. We first searched for a consensus sequence in the 5′ UTR of these top 20 loaded MS identify candidates but failed to identify such a sequence using a number of strategies (see Materials and Methods). Surprisingly, we also did not observe a difference in 5′-UTR length and GC content of these transcripts compared to poorly loaded transcripts (Fig. S1A and B). To determine if the 5′ UTR contributes to translational efficiency, a series of reporter gene constructs were made that differed only in their 5′ UTRs. To this end, we set up a luciferase (Lux) reporter lacking a 5′ UTR that is regulated by a 5X-NF-κB promoter (Fig. 5A) (28). The 5′ UTR from transcripts predicted to be translated either efficiently or inefficiently were then inserted upstream of the reporter, allowing the transcriptional fusions to be expressed specifically in response to L. pneumophila infection (pNL3.2.NF-κB-RE[NlucP/NF-κB-RE/Hygro]). In previous work, we demonstrated that the NF-κB promoter is quiescent in HEK293 cells in the absence of infection but becomes activated after exposure to L. pneumophila, dependent on the bacterial T4SS (28). This allowed us to determine the amount of translation specifically after challenge with L. pneumophila without the background contribution of translation before bacterial incubation. In a similar vein, as transcription from the reporter requires L. pneumophila incubation, quantitation of the *lux* transcript allowed accurate postinfection quantitation. This allows us to normalize the amount of protein synthesized to unit transcription.

Individual 5′ UTRs were selected from transcripts encoding candidates identified by AHA pulldown that were also predicted to have high levels of ribosome loading (Fig. 4C), and regions starting at the 5′ nucleotide and ending precisely at the nucleotide preceding the ATG were inserted directly upstream of the *lux* reporter gene start codon. As controls, 5′-UTRs from transcripts shown to have low ribosomal loading were selected (Fig. 4C). Each of the plasmids was transfected into HEK293-T cells, and after 24 h, cells were either harvested for further processing or challenged with L. pneumophila for 6 h. Luciferase activity was then measured as an indicator of mRNA translation, and the amount of *lux* transcript was determined by reverse transcription-quantitative PCR (qRT-PCR). As we have argued previously (28), HEK293 behaves very similarly to BMDMs with regard to the L. pneumophila translational block, as over 80% of the infected cells were shut down for translation, while a mutant lacking the T4SS (LP03) showed no translational interference (Fig. 5B). These results were very similar to data from BMDMs (29).

The luciferase activity normalized to transcript concentration was stable over a range of DNA concentrations in the transfection mix, indicating that the normalized activity was independent of transcript levels (Fig. 5C and D). The pNL3.2.NF-κB-RE[NlucP/NF-κB-RE/Hygro] with the native 5′ UTR was cotransfected with increasing amounts of a plasmid encoding the Nod1 protein, a known inducer of the NF-κB promoter. Total luciferase activity was found to be dependent on DNA concentration (Fig. 5C); however, when luciferase levels were normalized to transcript levels, there were no statistically significant differences between samples (Fig. 5D). A similar test was performed by challenging transfected cells with increasing amounts of L. pneumophila (Fig. 5E and F). Total luciferase activity was found to be a function of multiplicity of infection (MOI) (Fig. 5E); however, when normalized to Lux mRNA, luciferase activity was independent of the increasing amount of transcription that occurred in these samples (Fig. 5F). Therefore, luciferase activity normalized to transcription level is predicted to be an accurate measure of translation efficiency.

**FIG 6:**
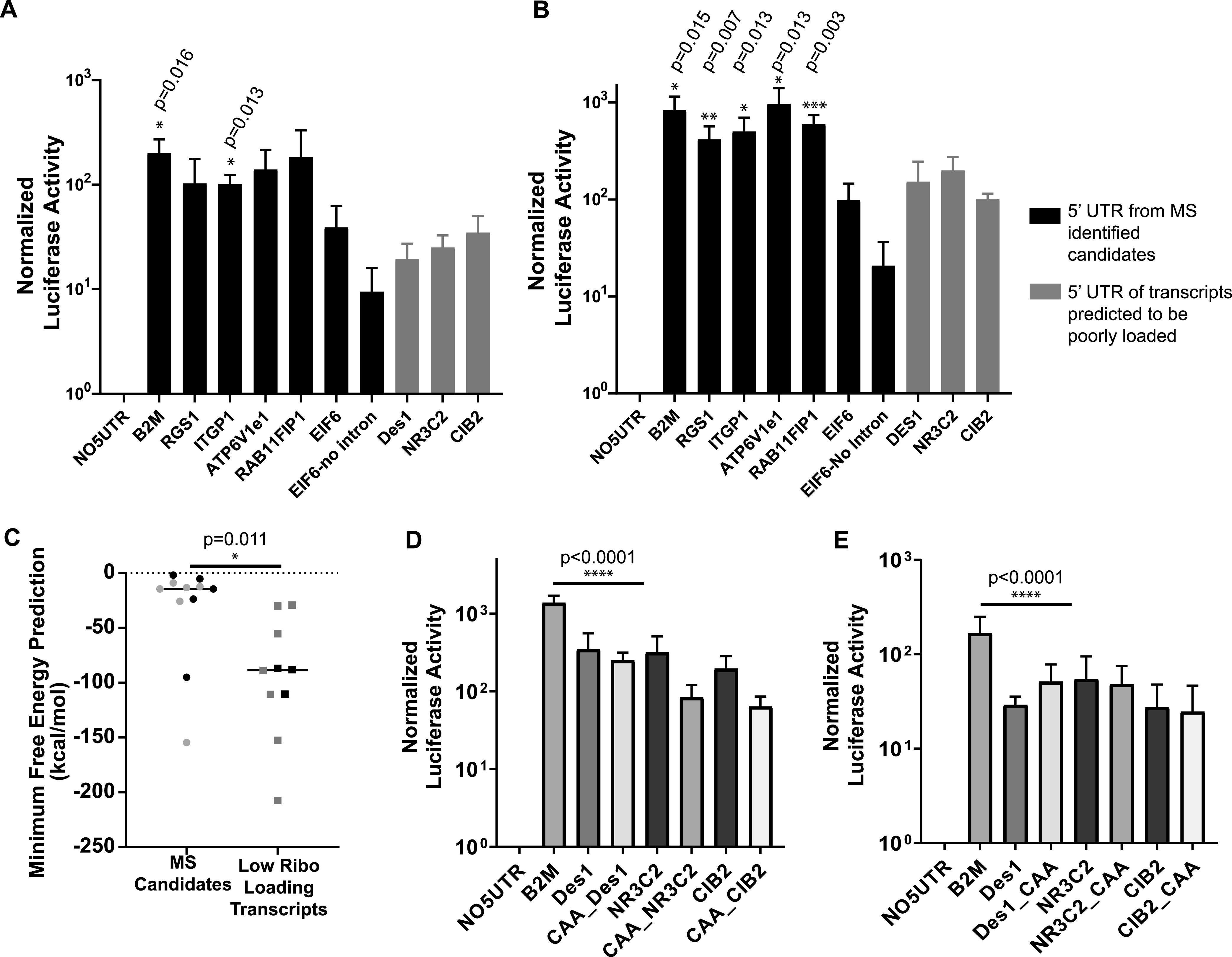
High ribosome loading transcripts have UTRs that enable efficient translation in the presence of L. pneumophila infection. (A) Luciferase activity after 24-h transfection with 1.6 μg of pNF-kBLUX plasmid containing selected 5′-UTR transcript (*n* = 3 biological replicates). qRT-PCR was performed to normalize data to the total mRNA levels of luciferase and β-actin. Black bars: 5′ end from candidates identified by MS. Gray bars: 5′ end of transcript predicted to be poorly (B) Transfectants having selected 5′-UTR transcripts were challenged by L. pneumophila (MOI = 25) and luciferase activity was determined after 6 h (*n* = 3 biological replicates). Data were normalized to total mRNA level of luciferase and β-actin, as determined by qRT-PCR. Black bars: 5′ end from candidates identified by MS. Gray bars: 5′ end of transcript predicted to be poorly loaded. (C) Predicted minimum free energies for experimentally tested 5′ UTRs (black) and 5′ UTRs with similar ribosomal loading efficiency as the experimentally validated 5′ UTRs (gray) (Materials and Methods) (76). (D and E) 5′ UTRs that drive low luciferase activity were modified with (CAA)_18_ at either the 5′ end of the transcript (D) or upstream of the translation start site (E), and constructs were transfected into HEK293T cells. The transfectants were then challenged by L. pneumophila for 6 h, and luciferase activities were determined (see Materials and Methods). Statistical analyses were performed on normalized data by unpaired *t* test (*, *P* < 0.05; **, *P* <0.01; ***, *P* < 0.001; see Materials and Methods).

To analyze the behavior of 5′ UTRs, we chose B2M (one of the most abundant MS candidates) and five of the AHA-pulldown candidates that were translated from low abundance transcripts having high ribosome loading after L. pneumophila infection of C57/BL6 BMDMs (Fig. 4C). As a control, three 5′ UTRs from transcripts that resulted in low-efficiency loading were chosen (Fig. 4C). The nine candidate 5′ UTRs were fused upstream of Lux (Fig. 5A), transfected into HEK293 cells, and challenged with L. pneumophila, assaying for luciferase activity both before and after infection. Prior to infection, it was clear that most of the MS candidates showed higher basal activity than the controls (Fig. 6A; underlying data; Data Set S5). After a 6-h infection, five of the six 5′ UTRs from transcripts encoding the MS candidates showed significantly higher normalized luciferase activities than the controls, which were defined as 5′ UTRs from transcripts that showed low-efficiency loading based on ribosome profiling, with differences ranging from 2 to 10 times greater than the controls (Fig. 6B; Data Set S5; *P* = 0.05 to 0.001, depending on the reporter). The one exception was the 5′ UTR of eIF6, which was among the MS candidates predicted to show high luciferase activity. The 5′ UTR of eIF6 is unique among this subset as it contains an intron, so to determine if the presence of the intron interfered with our ability to see enhanced translation, it was removed. However, the removal of the intron did not result in increased normalized luciferase activity, indicating that the translation efficiency of the eIF6 transcript may be controlled by features outside the 5′ UTR. We conclude that the structure of the 5′ UTR likely directs bypass of L. pneumophila translation inhibition in a large fraction of the transcripts encoding the MS candidates, but a subset of transcripts exist with other mRNA features that can facilitate bypass. Furthermore, the bypass is an intrinsic characteristic of the 5′ end of the transcript and not dependent on infection, as luciferase activities from transcripts encoding MS candidates were higher than the low-loading controls even in the absence of L. pneumophila challenge or when challenged with LPO3 (Fig. S2).

As the 5′-UTR secondary structure has been proposed to play an essential role in regulating translational efficiency (53–55), we examined predicted secondary structures of each transcript variant that encodes the above-described experimentally validated 5′ UTRs (Fig. 6C, black points) and 5′ UTRs with similar loading efficiency as the experimentally validated 5′ UTRs (Fig. 6C, gray points). Based on the Vienna RNA secondary structure algorithm, folding structures were predicted, as were the corresponding free energy changes (56). The minimum free energy for RNA folding of mRNA 5′ UTR for this subset of genes was found to be significantly higher than poorly loaded transcripts, indicating that the poorly loaded transcripts likely have areas with an extensive secondary structure not found in most of the efficiently translated transcripts (Fig. 6C). These results are consistent with unstructured 5′ UTRs conferring a translational advantage to transcripts in L. pneumophila-infected cells.

As 5′-UTR structures appear to regulate translational efficiency during L. pneumophila challenge of BMDMs, we manipulated the 5′ UTR of the subset of transcripts that were poorly translated. Initiation factor eIF4F is recruited to the 5′ leader cap and collaborates with eIF4A to unwind the mRNA allowing for docking of the 40S ribosomal subunit (57). Steric barriers in the 5′ leader region can decrease translational efficiency, but it has been found that adding sequences lacking secondary structures either upstream or downstream of these structures, such as [CAA]_n_ repeats, can increase translational efficiency (58–61). We inserted [CAA]_18mer_ either proximal to the 5′-UTR cap (Fig. 6D; Data Set S5) or upstream of the translation start site (Fig. 6E; Data Set S5). Insertion of these unstructured 5′-UTR sequences was not sufficient to alleviate the translation suppression induced by the predicted secondary structures of these 5′ UTRs during L. pneumophila challenge (Fig. 6C and D). Therefore, if secondary structure blockade interferes with ribosomal subunit docking, initiation of scanning by the preinitiation complex cannot overcome this blockage.

## DISCUSSION

A number of pathogens encode proteins that interfere with host cell translation, leading to host cell misregulation, disruption of tissue organization and stimulation of innate immune signaling (23–26, 62). The reasons for exerting translational control are varied, and range from supporting microbial colonization through localized inflammation to converting host translation into viral manufacturing platforms (63). L. pneumophila is one such translation-blocking pathogen (22, 29, 64). The mechanisms by which host cells can mount an immune response to restrict pathogen growth after translation blockade are poorly understood. In the case of host cellular responses to Pseudomonas aeruginosa and L. pneumophila, protein synthesis inhibition is tightly linked to transcriptional activation of antimicrobial signaling pathways, which provide the pool for residual translation in response to the pathogen. Recognition of microbial pattern molecules is central to this activation, as TLR signaling via MyD88 activates transcription, allowing bypass of protein synthesis inhibition (29, 36). In addition, bystander cells, sensing either liberated microbial fragments or low-level cytokine production from infected cells, amplify this response and appear to be the primary source of inflammatory cytokine production in tissues (35).

In this work, we determined if selective bypass of translational inhibition could be explained exclusively by transcriptional hyperinduction, resulting in the secretion of innate immune effectors (29, 36). To test this model, we identified the most abundant proteins synthesized from 4 to 6 h after L. pneumophila infection of macrophages, using snapshot proteomics analysis of the infected cell subpopulation. Among the over 300 identified proteins, a large number were synthesized from the most abundant macrophage transcripts (Fig. 4C), many of which required MyD88 for hyperinduction (Fig. 3C). This supports previous arguments regarding transcriptional hyperinduction (20) but does not fully explain *de novo* protein production. Proteins synthesized from transcripts that showed little or no induction in response to L. pneumophila were readily identified (Fig. 2D), and mutational inactivation of innate immune transcription driven by either type I IFN (Fig. 2B) or MyD88 recognition (Fig. 3C) did not prevent many of these proteins from being synthesized after bacterial infection. Most significantly, there was a group of proteins that were translated from poorly transcribed genes (Fig. 4D). Therefore, we believe that there are determinants other than transcriptional hyperactivation that contribute to bypass of L. pneumophila-dependent translation inhibition.

Our results provide evidence for translational control mechanisms that combat pathogen-directed inhibition of protein synthesis. Many of the proteins we identified after L. pneumophila challenge were derived from poorly transcribed, highly ribosome-loaded transcripts (Fig. 4C and D) (20). Surprisingly, although translational control of immune-related transcripts is potentially an important host strategy to combat pathogen attack, there has been minimal investigation of how the host uses ribosome loading to combat pathogen growth. Translation blockade could provide a significant hurdle for the pathogen, as viruses particularly are dependent on the host translation machinery for their replication (65). Therefore, they must either overwhelm the host with transcripts or evolve strategies to allow efficient translation of viral transcripts. It is logical to assume that the host has coevolved mechanisms that similarly confer a translation advantage of immune-related transcripts in the face of translational attack. In particular, ISGs represent the first line of cellular defense against pathogens. Interestingly, we found that most of the proteins identified that were selectively synthesized after L. pneumophila attack are encoded by ISGs.

Preferential translation of a subset of transcripts can be explained by a number of molecular strategies that facilitate the expression of immune response genes in the face of pathogen-encoded protein synthesis inhibitors. For example, it has been shown that translation efficiency could be altered by RNA structural elements such as upstream open reading frames (uORFs) or internal ribosome entry sites (IRES) (66). The binding of translational regulatory proteins or noncoding RNAs within the 5′ end of transcripts also has the potential to modulate translation rates (67). We argue, however, that in the presence of L. pneumophila challenge the secondary structure of the 5′ UTR is likely to be the important determinant that controls translation rates. The frequency of uORFs or IRES elements appeared to be approximately the same in either efficiently loaded, poorly expressed, or total cellular transcripts (Fig. S3 and 4). In contrast, folding algorithms interrogating the 5′ UTR indicated that lack of secondary structure positively correlated with efficient translation (Fig. 6). This prediction was strongly supported by our analysis of 5′-UTR fusions to a heterologous reporter, as MS candidates encoded by poorly transcribed genes had 5′ UTRs that resulted in efficient luciferase production and lower secondary structure. The one exception to this rule, in which the 5′ UTR of eIF6 was not sufficient to drive high luciferase production, argues that protein-coding sequences can sometimes drive efficient protein synthesis in the presence of *Legionella* translation inhibitors. In fact, secondary structures located within the coding region have been shown to increase efficient loading of the ribosomal 43S preinitiation complex at the start codon, providing an alternate strategy that could explain efficient translation of eIF6 (58).

Our data argue that translation efficiency is based on the nature of the 5′ UTR, and differential efficiency is maintained either in the presence or absence of protein synthesis inhibition by L. pneumophila translocated effectors. These data do not argue that certain classes of transcripts are selectively downmodulated. In fact, many transcripts in which there is inefficient translational loading are predicted to produce large amounts of protein due to high levels of transcription in response to bacterial infection (Fig. 4). This point is emphasized by our use of the NF-κB reporter system, which allows poorly translated transcripts to increase their total load of protein after challenge with L. pneumophila (Fig. 5). In the presence of infection, the NF-κB promoter is highly upregulated resulting in large amounts of transcripts. To allow us to measure relative translation efficiencies under conditions of this upregulation, normalization to transcription levels was required to determine if preferential translation occurs due to the presence of an activating 5′-UTR controlling element, which is the only variable in the luciferase reporter readout experiment.

Previous work indicated that inhibition of translation by 5′-UTR secondary structures can be reversed by increasing their distance from the 5′ end of the mRNA. This can be accomplished by inserting sequences lacking secondary structure, such as CAA repeats, between the 5′ end and the element having high secondary structure (68). The insertion of unstructured upstream regions is thought to facilitate scanning of the 5′ UTR by the 43S preinitiation complex, allowing the AUG start codon to be identified (69). Given that we found a strong correlation between unstructured 5′ UTRs and high translation levels, it is surprising that the addition of CAA repeats at the 5′ end of the poorly translated mRNAs failed to enhance translation efficiency (Fig. 6D and E). This result argues that the 5′-UTR secondary structures found in poorly translated mRNAs may present profound blocks on scanning even under conditions in which scanning is efficiently initiated at the 5′ end. In support of this model, inhibitory secondary structure elements experimentally shown to be reversed by CAA repeats have calculated Δ*G* values of −30 to −50 kCal/mol (68), while most of the inefficiently translated 5′ UTRs in our study are predicted to have Δ*G* values that are considerably lower (Fig. 5). Alternatively, L. pneumophila infection could interfere with the function of translation initiation factors, preventing efficient movement through secondary structure elements after initial scanning of unstructured regions (33, 36).

In summary, this work provides evidence that efficient loading of low-abundance transcripts is potentially an arm of the host defense against microbial infection. Many of these efficiently translated transcripts are interferon-regulated and/or fail to be induced in cells targeted by L. pneumophila. These results support previous work showing that low-level transcription of antimicrobial factors is an important strategy for defending against pathogens that block translation or block innate immune response pathways in host cells (70). Infected cells can then liberate either degraded microbial products or cytokines that signal to uninfected bystander cells to amplify the anti-pathogen response (35). As pathogens are proficient at interfering with their recognition by host cells, this strategy is likely to be an important tool used by the host in the arms race with the pathogen.

## MATERIALS AND METHODS

### Ethics statement.

This study was carried out in accordance with the recommendation in the *Guide for Care and Use of Laboratory Animals* of the National Institutes of Health. The Institutional Animal Care and Use Committee of Tufts University approved all animal procedures. The approved protocol number is B2013-18. The animal work, which is limited to macrophage isolation, did not involve any procedures that infected live animals.

### Cell culture.

L-cell supernatants were generated as described previously (71). BMDMs were from the femurs and tibias of female C57BL/6J as well as congenic MyD88^−/−^ and IFNAR^−/−^ mice (Jackson Laboratory, Bar Harbor, ME, USA). BMDMs were differentiated for 7 days in Roswell Park Memorial Institute (RPMI; Gibco) medium containing 30% L-cell supernatant, 10% fetal bovine serum (FBS), 1 mM l-glutamine, and 1× penicillin/streptomycin solution (100 U/mL penicillin, 100 μg/mL streptomycin) with feeding on day 4 of incubation. Cells were replated in antibiotic-free RPMI (10% L-cell supernatant, 10% FBS, 1 mM l-glutamine) medium 24 h before infection with L. pneumophila. HEK293 cells (ATCC CRL-1573) were grown in Dulbecco’s modified Eagle’s medium (DMEM; Gibco) supplemented with 10% heat-inactivated fetal bovine serum (FBS).

### Bacterial strains and infection.

All L. pneumophila strains are derived from LP02 [14], which is a streptomycin-resistant thymidine auxotroph. LPO2 Δ*flaA*-GFP^+^ (referred to as WT) and LPO3 Δ*flaA*-GFP^+^ (referred to as *dotA3*) carry GFP on an isopropyl-β-d-thiogalactopyranoside (IPTG)-inducible, Cm-resistant plasmid (*ori*RSF101Cam^R^p*tac*::GFP^+^; referred to as pGFP). Strains containing the pGFP plasmid were maintained on BCYE plates (72) containing 100 μg/mL thymidine and 5 μg/mL chloramphenicol and grown in AYE broth (72) containing 100 μg/mL thymidine, 5 μg/mL chloramphenicol, and 1 mM IPTG.

For experiments involving challenge of cultured cells with bacteria, L. pneumophila colonies were patched on BCYE plates, and 36 h later, 2-fold dilutions of L. pneumophila strains were grown overnight in AYE broth culture at 37°C with shaking. Cultures were grown to postexponential phase (A_600_ of 3.8 to 4.5), and dilution in this range was selected for challenge of mammalian cells. BMDMs were plated at a density of 1.56 × 10^5^ cells per cm^2^, and BMDMs were challenged at various MOIs (assuming that A_600_ = 1.0) is equivalent to 10^9^ bacteria/mL. Contact was initiated by centrifugation for 10 min at 400 RCF, and 1 h postinfection, the medium was changed, and cells were maintained in the appropriate medium supplement with 200 μg/mL of thymidine.

### Metabolic labeling and quantification.

For all metabolic labeling experiments, BMDMs were plated at 1.56 × 10^5^ cells per cm^2^ on Costar Clear-Not-Treated 6-well plates (Corning; 3736) with RPMI containing 10% L-cell supernatant and 10% FBS. After 24 h, the medium was changed to serum-free, methionine-free RPMI medium 1 h before infection. Cells were challenged with L. pneumophila at MOI of 15 by centrifugation for 10 min at 400 RCF. After 1 h, the cultured medium was changed to fresh RPMI (methionine and serum-free), and 50 μM AHA (Invitrogen) was added to the medium at noted time points for a minimum of 1 h incubation. Cells were harvested at the indicated time points by placing them on ice and washing them twice with cold PBS. After the second wash, cells were harvested in cold PBS, pelleted, and fixed with 4% PFA for 15 min. Fixed cells were washed twice with PBS and blocked with BSA/PBS for 1 h. After 1 h, 100 μM APC-conjugated phosphine reagent (Pierce) in blocking buffer was added. Cells were incubated at 37°C for 2 to 3 h and washed three times with 0.5% Tween 20/PBS followed by analysis on a Becton-Dickenson FACScalibur.

### Identification of newly synthesized proteins by click chemistry and mass spectrometry.

To identify newly synthesized proteins in response to L. pneumophila challenge, BMDMs were plated at 1.56× 10^5^ cells per cm^2^ on Costar Clear Not Treated 6-well plates (Corning, 3736) with RPMI containing 10% L-cell supernatant and10% FBS. Cells were changed to serum-free, methionine-free RPMI medium 1 h before infection, then challenged with L. pneumophila Δ*flaA*-GFP^+^ at MOI = 15. The medium was then changed to fresh methionine-free RPMI 1 h postinfection followed by addition of 100 μM AHA at 4 h postinfection. After 2 h further incubation, cells (2.4 × 10^7^) were then placed on ice, lifted in 2 mL cold PBS by pooling two 6-well plates and sorted by BD FACSAria. Then, 1 × 10^7^ GFP^+^ cells were the collected, pelleted, and flash frozen for further proteomic analysis.

Pulldown of newly synthesized proteins using the Click-iT Protein Enrichment kit followed manufacturer’s protocols (Thermo-Fisher). The flash-frozen cells were lysed by resuspending in Urea lysis buffer (8 M Urea, 200 mM Tris pH 8.0, 3% CHAPS, and 1 M NaCl), supplemented with protease inhibitors, and then treated with Benzonase (2.4 U/mL) followed by incubation on ice for15 to 30min. The lysate was then vortexed for 5 min, debris was pelleted at 10,000 RCF for 5 min, and the supernatant was transferred to a fresh tube for further analysis.

To set up the Click Reaction, 2× catalyst solution was mixed with the 800 μL of cell lysate and 200 μL of resin slurry. The sample was rotated end-over-end at room temperature for 20 h, resin was pelleted by 1 min centrifugation at 1,000 RCF, and the supernatant was removed. One milliliter of SDS wash buffer (Click-iT Protein Enrichment kit) containing 10 μL of 1 M DTT was added to the resin and then heated to 70°C for 15 min before incubation at room temperature for 15 min. The resin was pelleted for 5 min at 1,000 RCF, and the supernatant was removed. The 1-mL resin-bound protein fraction was then incubated with the addition of 7.4 mg of iodoacetamide and incubated in the dark for 30 min. The resin was pelleted, and transferred to a column for washing. The column containing the beads was washed 5× with SDS wash buffer (Click-iT Protein Enrichment kit), 5× with 8 M urea/100 mM Tris pH 8, 5× with 20% acetonitrile in water. To digest the resin-bound protein, the resin was resuspended in 500 μL of digestion buffer (100 mM Tris-HCl pH 8, 2 mM CaCl_2_, 10% acetonitrile). Then 0.1 μg/μL of trypsin was added to the resin slurry, and the mixture was incubated at 37°C overnight. After the overnight digestion, the resin was pelleted by centrifugation for 5 min at 1,000 RCF, the supernatant was collected, and the samples were acidified with 2 μL of trifluoroacetic acid to stop further reaction.

Samples were submitted to the Taplin Mass Spectrometry Facility (https://taplin.med.harvard.edu/home) for LC-MS/MS. To account for the mass gain due to incorporating the methionine analog, AHA sample parameters were modified to account for either a 107 atomic mass unit gain if the analog is incorporated instead of methionine or the incorporation of endogenous methionine. The LC-MS/MS was performed in duplicate, and the results were analyzed using a modified Z-score to rank-order candidates. Other strategies, such as individual peptide abundance, gave similar results. To determine the *z*_MOD,_ the deviation of each protein from the median intensity of proteins in the sample was determined as the Median Absolute Deviation (MAD). From this calculation, the *z*_MOD_ was determined as described (73) and outliers were determined as *z*_MOD_ ≥3.5. Outliers were then interrogated using Ingenuity Pathway Analysis (IPA) database (Qiagen Inc.; https://digitalinsights.qiagen.com) which categorizes proteins based on function, pathway, and network. Candidates from the LC-MS/MS analysis were further dissected using Interferome (http://interferome.its.monash.edu.au/interferome/home.jspx; version 2.01).

### Immunoblot analysis of protein levels in infected BMDMs lysates.

BMDMs were plated at 1.56× 10^5^ cells per cm^2^ on Costar Clear Not Treated 6-well plates with RPMI containing 10% L-cell supernatant/10% FBS. After 24 h, cells were challenged with L. pneumophila at MOI of 15, followed by contacting the BMDMs in the centrifuge at 400 RCF for 10 min. At the indicated time points, the medium was removed and adherent cells were lifted with ice-cold PBS and pelleted at 1,000 RCF for 5 min in the centrifuge. Cells were sorted and lysed with 1 × SDS Laemmli sample buffer (0.125 M Tris-Cl pH 6.8, 4% SDS, 20% glycerol, 10% beta-mercaptoethanol, 0.01% bromophenol blue). Cell lysates were boiled for 5 min and fractioned on SDS-polyacrylamide gels (Bio-Rad) and then electroblotted on nitrocellulose membranes. Blots were blocked with 5% BSA in Tris-buffered saline-tween (TBST: 0.05 M Tris-HCl pH 8.0, 0.138 M NaCl, 0.0027 M KCl, 0.05% Tween 20). For immune detection, cells were probed with a primary antibody (1:1,000 or manufacturer’s recommendation) in 5% BSA/TBST overnight at 4°C. After being washed with TBST, secondary antibodies Dylight anti-rabbit IgG 680, Dylight anti-mouse IgG 680, Dylight anti-rabbit IgG 800, or Dylight anti-mouse IgG 800 (Cell Signaling 1:20,000) were incubated in 4% milk/TBST for 45 min at room temperature. The membranes were scanned by Odyssey Imaging System and the Image Studio software (LI-COR Biosciences).

### Processing of sequencing data.

BMDMs from WT and MyD88^−/−^ mice were challenged with LPO2 Δ*flaA*-GFP^+^ at MOI of 3 and cells harboring bacteria were sorted by flow cytometry based on GFP fluorescence (Fig. 3A to D). RNA was extracted from sorted cells, treated with DNase (Turbo DNA-free kit; Invitrogen), and used for generating RNA-Seq library using TruSeq stranded total RNA library prep kit (Illumina). cDNA fragments from the library were sequenced by Illumina HiSeq 2000 (150 bp, single-end reads). RNA sequencing reads were processed using CLC Genomics Workbench (Qiagen). Reads were preprocessed by first trimming the linker sequence from the 3′ end and then aligned to the mouse genome (mm10). For read mapping parameters, a maximum of four mismatches were allowed, and multimapping of up to eight different positions was permitted. mRNA transcription track alignment was performed. Only one genomic position per alignment was allowed. The RPKM value was calculated as the expression value. RNA-seq data were deposited in BioSample (SAMN10180267, SAMN10180268). RNA-seq and Ribo-seq data for L. pneumophila-challenged C57BL/6 BMDMs were also obtained from the published data set GSE89184 (Fig. 4) (36).

### 5′-UTR sequence analysis.

5′-UTR sequences of selected transcripts were obtained from the Ensemble genome browser (http://useast.ensembl.org/index.html; Ensembl Genes 103, GRCm39) in FASTA format. In the cases in which more than one transcript was available, the transcripts with the highest ribosomal loading were chosen. The MEME algorithm present at the MEME suite database (University of Nevada, Reno, University of Washington, Seattle, WA, USA; version 5.3.3; http://meme-suite.org/) was used for identification of sequence motifs in a collection of unaligned nucleotide sequences (70). Possible uORF and IRES sequence features were analyzed by comparing them to a publicly available uORF-containing data set (74) and IRES-containing data set (75). GC content was calculated as a percentage-based formula: Count(G + C)/Count(A + T + C + G) × 100. Secondary structure and minimum free energy were predicted by RNAfold (Institute for Theoretical Chemistry, University of Vienna, Vienna, Austria; http://rna.tbi.univie.ac.at/cgi-bin/RNAWebSuite/RNAfold.cgi) (76).

### Construction of reporter plasmids.

A sequence spanning the transcription start site to the translation start site of the selected 5′ UTRs was amplified by PCR from mouse BMDM genomic DNA using the primers listed in Table S1, containing flanking sequences that matched the pNL3.2NF-κB-RE plasmid (N111A; Promega). The pNL3.2NF-κB-RE plasmid was PCR amplified with primers containing sequences flanking the 5′ UTR. Amplification was carried out in a PCR Thermal Cycler (Thermo Scientific) with a preliminary denaturation step at 94°C for 5 min, followed by 30 cycles at 94°C for 45 s, primer annealing at 60°C for 15 s and primer extension at 72°C for 30 s, followed by a 2-min final extension at 72°C. PCR products were cleaned using QIAquick Gel Extraction Protocol (Qiagen). The PCR fragment and the vector were gel extracted and combined in a Gibson Assembly Reaction (NEB, E2611S) and transformed into DH5α. Clones were sequenced, and positive clones were stored at −80°C.

### 5′ UTR luciferase activity reporter measurements during L. pneumophila challenge.

HEK293 cells were plated at 1 × 10^5^ on 12-well plates in DMEM (10% FBS). Twenty-four hours after seeding, cells at ~80% confluence were subject to transfection using Lipofectamine 2000 reagent (Invitrogen) according to the manufacturer’s protocol. The cells were either transfected with the indicated concentration of pNL3.2NF-κB-RE plasmid reporter construct alone or cotransfected with pMYCNOD1. Twenty-four hours posttransfection the medium was replaced, and cells were challenged L. pneumophila at the indicated MOI followed by 400 RCF centrifugation for 10 min. After 1 h, the medium was replaced, and the cells were incubated for another 5 h. Cells were harvested 6 h postinfection with cold PBS. Two washes with PBS were performed, and the cells were resuspended in1ml of cold PBS. The sample was split into two aliquots; 100-μL aliquots were used for luciferase measurements, and 900-μL aliquots were used for RNA extract and qRT-PCR.

Luciferase activity was quantified using the Nano-Glow Luciferase Assay System (Promega) according to the manufacturer’s instructions. Then, 100-μL aliquots were transferred to a Corning 96-well White Flat Bottom polystyrene plate for luciferase measurements. Luminescence was measured using the Synergy Microplate reader (BioTek Instruments) and was determined as relative luminescence units. Briefly, one volume of Nano-glow luciferase assay reagent equal to the sample volume was added. The mixture was incubated for 3 min, and the luminescence intensity was measured. To correct for differences in transfection efficiency, luciferase activities were normalized to luciferase mRNA transcript values and β-actin transcript values in each sample.

For total RNA preparation, to determine luciferase activity, 900-μL cell samples were pelleted and RNA was extracted from cells by using the RNeasy kit (Qiagen). The resulting total RNA sample was diluted to 1 μg of total RNA in 10 μL of H_2_O and treated with ezDNASE (Invitrogen) enzyme to digest gDNA. cDNAs were synthesized using SuperScript IV VILO Reverse Transcriptase kit (Life Technologies) with random primers using 1 μg of RNA as a template. Each cDNA sample was used as a template to analyze luciferase transcript levels using primers in Table S3 in the supplemental material. The expression level of luciferase was standardized by normalizing it to the expression levels of β-actin. SYBR green PCR Master Mix reagent was used to perform quantitative PCR.
