# Supplementary material for: 5′ Untranslated mRNA Regions Allow Bypass of Host Cell Translation Inhibition by Legionella pneumophila
Source: Infect Immun. 2022 Nov 2;90(11):e00179-22. doi: 10.1128/iai.00179-22 (PMC9670960; doi:10.1128/iai.00179-22)
Supplement: Supplemental file 6 — Table S1 and Fig. S1 to S4. Download iai.00179-22-s0001.pdf, PDF file, 6.2 MB [file iai.00179-22-s0001.pdf]

Supplementary Table 1: Plasmids and Primers used in this study

Plasmid	Description	Source
pNL3.2NF-κB-RE	NanoLuc Reporter Vector with NF-κB RE	Promega
**Primer Name**	**Primer Sequence (5'-3')**	**Description**
**Primers of qPCR analysis**		
QF-pNLRT	CAG GGA GGT GTG TCC AGT TT	Quantify expression of Luciferase gene
QR-pNLRT	GCC ATA GTG CAG GAT CAC CT	
QF-B-Actin	ACA GAG CCT CGC CTT TGC C	Quantify expression of β-Actin gene
QR-B-Actin	GAT ATC ATC ATC CAT GGT GAG CTG G	
**Primers for Cloning**		
pNLUX_ITGB1_fwd	CTG CGA AAA GAT GGT CTT CAC ACT CGA AG	Vector Amplification for ITGB1 Cloning
pNLUX_ITGB1_rev	TCA GCG AGT GCT GGA AGT CGA GCT TCC ATT ATA TAC	
ITGB1_fwd	CGA CTT CCA GCA CTC GCT GAA GAG CCG C	5'UTR Amplification for ITGB1 Cloning
ITGB1_rev	TGA AGA CCA TCT TTT CGC AGC GTC CGC C	
RGS1_FWD	CTC ACT CGT TTT GAG AAG ACC ATG GTC TTC ACA CTC GAA G	Vector Amplifiction for Insertion RGS1 5'UTR
RGS1_REV	GGT CTT CTC AAA ACG AGT GAG CTG GAA GTC GAG CTT CCA TTA TAT AC	
pNLUX_Nr3C2_fwd	CAA GGC AGC TAT GGT CTT CAC ACT CGA AG	Vector Amplification for Nr4C2 Cloning
pNLUX_Nr3C2_rev	GCT GAG GGG ACT GGA AGT CGA GCT TCC ATT ATA TAC	
Nr3c2_fwd	CGA CTT CCA GTC CCC TCA GCT CCT GCG C	5'UTR Amplification for Nr3c2 Cloning
Nr3c2_rev	TGA AGA CCA TAG CTG CCT TGG CCA GGG T	

pNLUX_ATP6V1e1_fwd	TGC CTT CGC CAT GGT CTT CAC ACT CGA AG	Vector Amplification for ATP6V1e1 Cloning
pNLUX_ATP6V1e1_rev	AAC CTG AGA CCT GGA AGT CGA GCT TCC ATT ATA TAC	
ATP6V1E1_fwd	CGA CTT CCA GGT CTC AGG TTT GCT CCG C	5'UTR Amplification for ATP6V1e1 Cloning
ATP6V1E1_rev	TGA AGA CCA TGG CGA AGG CAA AGT CCG G	
pNLUX_eIF6_fwd	CAT CGG GCG CAT GGT CTT CAC ACT CGA AG	Vector Amplification for eIF6 Cloning
pNLUX_eIF6_rev	TGG TCA CTC TCT GGA AGT CGA GCT TCC ATT ATA TAC	
eIF6_fwd	CGA CTT CCA GAG AGT GAC CAG CAG CTC C	5'UTR Amplification for eIF6 Cloning
eIF6_rev	TGA AGA CCA TGC GCC CGA TGA TTC GGC A	
pNLUX_Cib2_fwd	CGC GGT CAC CAT GGT CTT CAC ACT CGA AG	Vector Amplification for Cib2 Cloning
pNLUX_Cib2_rev	GGG AGG CAA ACT GGA AGT CGA GCT TCC ATT ATA TAC	
Cib2-201_fwd	CGA CTT CCA GTT TGC CTC CCA GCC CCT G	5'UTR Amplification for Cib2 Cloning
Cib2-201_rev	TGA AGA CCA TGG TGA CCG CGA TGG TAT GG	
pNLUX_Rab11fip1_fwd	GTC TGC CAC CAT GGT CTT CAC ACT CGA AG	Vector Amplification for Rab11fip1 Cloning
pNLUX_Rab11fip1_rev	GGT CTG GAC CCT GGA AGT CGA GCT TCC ATT ATA TAC	
Rab11fip1-203_fwd	CGA CTT CCA GGG TCC AGA CCG AGG ACA GCG	5'UTR Amplification for Rab11fip1 Cloning
Rab11fip1-203_rev	TGA AGA CCA TGG TGG CAG ACG CGG CAC C	
pNLUX_DESI1_fwd	ACG GGT CCT GAT GGT CTT CAC ACT CGA AG	Vector Amplification for DESI1 Cloning
pNLUX_DESI1_rev	TGA CAG AGA GCT GGA AGT CGA GCT TCC ATT ATA TAC	
DESI1_fwd	CGA CTT CCA GCT CTC TGT CAC CTC ACA GTA GTC GTT GCC	5'UTR Amplification for DESI1 Cloning

DESI1_rev	TGA AGA CCA TCA GGA CCC GTG GCG GCG G	
pNLUX_eIF6_fwd	CAT CGG GCG CAT GGT CTT CAC ACT CGA AG	Vector Amplification for eIF6 Cloning
pNLUX_eIF6 _rev	TGG TCA CTC TCT GGA AGT CGA GCT TCC ATT ATA TAC	
EIF6_1_fwd	CGA CTT CCA GAG AGT GAC CAG CAG CTC C	5’UTR Amplification for eIF6 Cloning
EIF6_1_rev	CTC GAA CAC ACA AGC GAC TCC AAT GTC TAC	
EIF6_2_fwd	GAG TCG CTT GTG TGT TCG AGG GTG AGC TC	5’UTR Amplification for eIF6 without intron Cloning
EIF6_2_rev	TGA AGA CCA TGC GCC CGA TGA TTC GGC A	
DES1_FWD-CAAS2	AGC TCG ACT TCC AGA ACA ACA ACA ACA ACA ACA ACA ACA ACA ACA ACA ACA ACA ACA ACA ACA ACA ACA ACA ACT CTC TGT CAC CTC ACA GTA GTC GT	Vector Amplification for DESI1 5’UTR CAA_{18mer} Cloning
NR3C2_FWD_CAAS2	CGA CTT CCA GAA CAA CAA CAA CAA CAA CAA CAA CAA CAA CAA CAA CAA CAA CAA CAA CAA CAA CAA TCC CCT CAG CTC CTG CGC ACC CG	Vector Amplification for NR3C2 5’UTR CAA_{18mer} Cloning
CIB2_FWD_CAAS2	CGA CTT CCA GAA CAA CAA CAA CAA CAA CAA CAA CAA CAA CAA CAA CAA CAA CAA CAA CAA CAA CAA TTT GCC TCC CAG CCC CTG CAC CCC T	Vector Amplification for CIB2 5’UTR CAA_{18mer} Cloning
REV_CAAS2	GTT GTT GTT GTT GTT CTG GAA GTC GAG CTT CCA TTA TAT AC	Vector Amplification for 5’UTR CAA_{18mer} Cloning

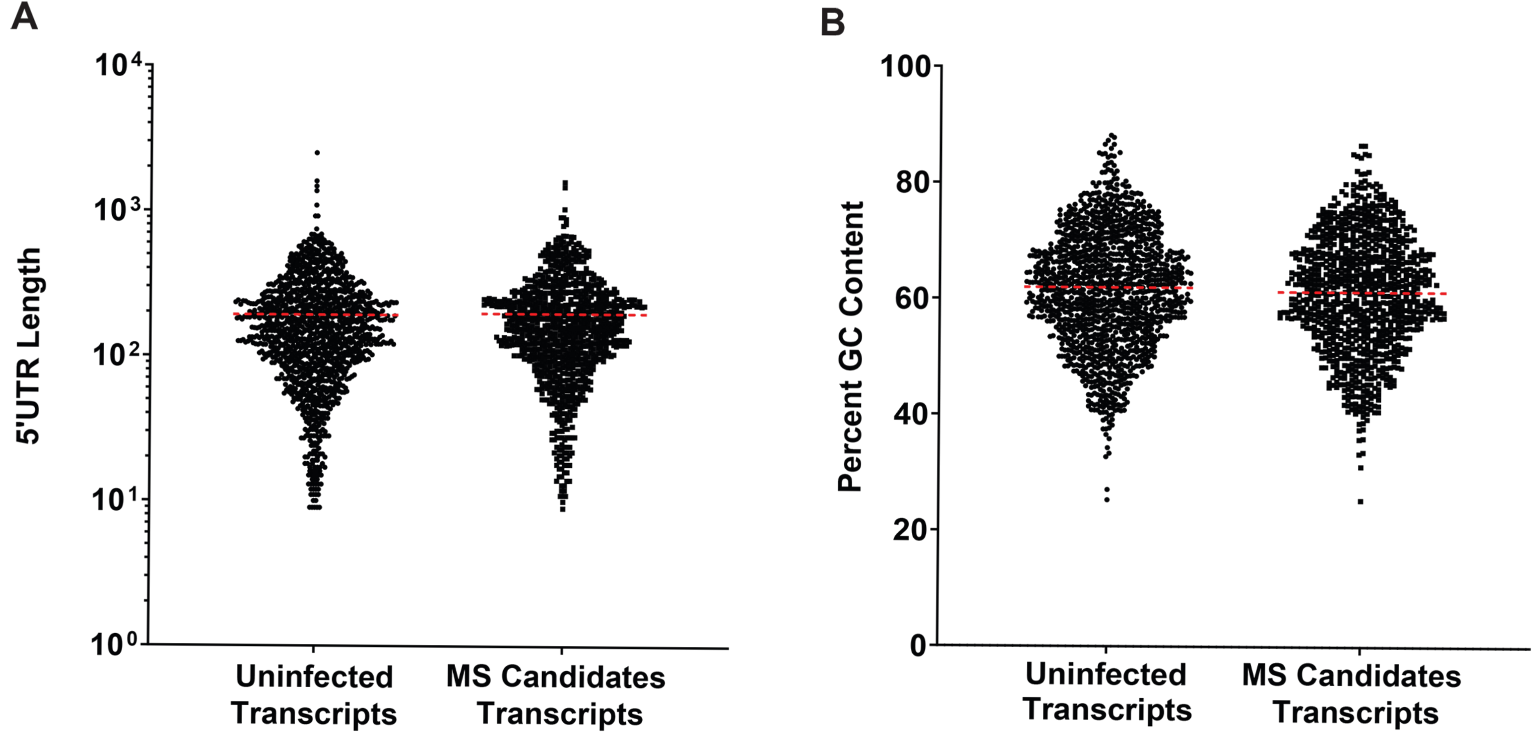

Supplementary Figure 1. Distribution of Length and GC content of 5'UTR of MS candidates is unaltered relative to uninfected controls. (A-B) Comparison of the RefSeq-annotated 5'UTR lengths (A) and GC content (B) for transcripts of proteins identified by MS. The dot plots depict the distribution of 5'UTR length and GC content with no statistical significance between samples. Unpaired t-test statistical analyses was performed.

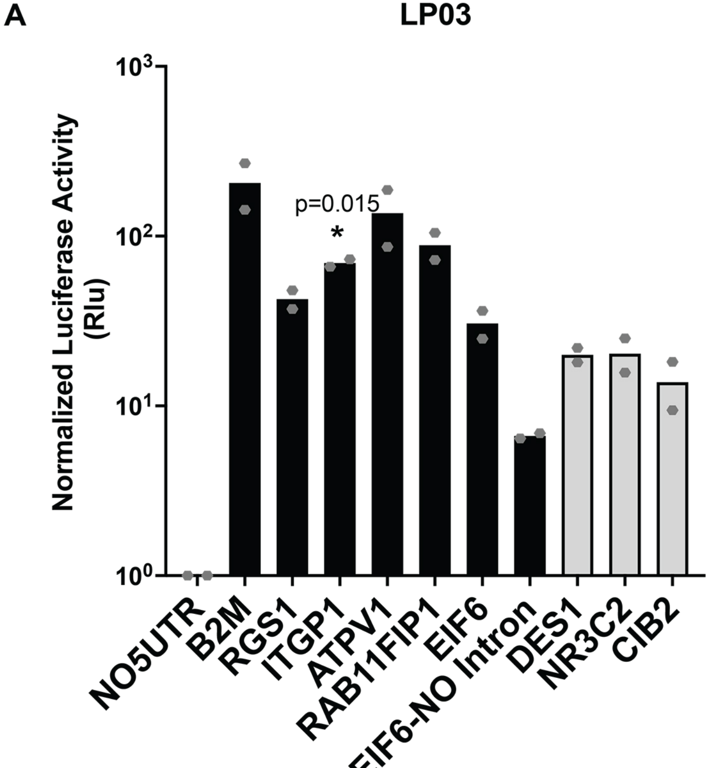

Supplementary Figure 2. High ribosome loading transcripts have UTRs that enable efficient translation in the presence of L. pneumophila LPO3 infection (A). Transfectants having selected 5'UTR transcripts were challenged by L. pneumophila LPO3 (MOI=25) and luciferase activity was determined after 6 hr (n=2 biological replicates). Data were normalized to total mRNA level of luciferase and β-Actin, as determined by qRT-PCR. Black bars: 5' end from candidates identified by MS. Gray bars: 5' end of transcript predicted to be poorly loaded.

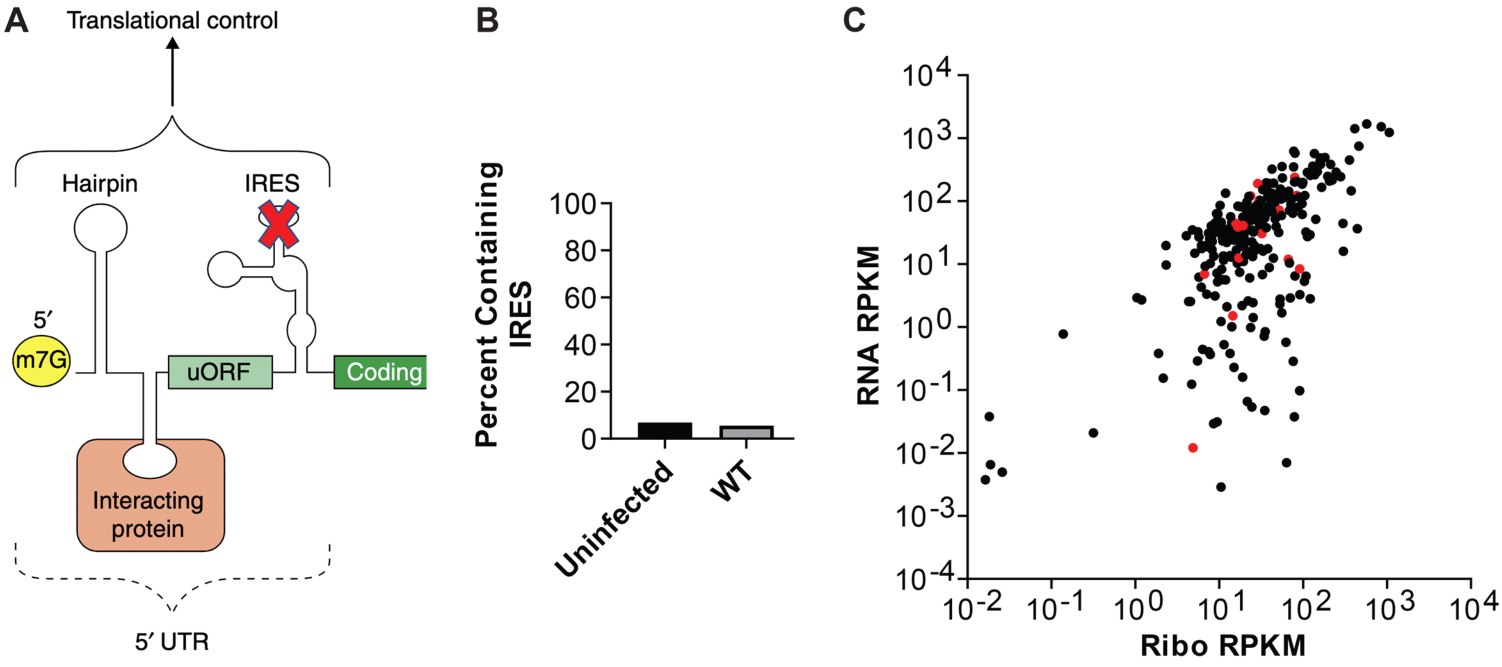

Supplementary Figure 3. IRES are not enriched in MS candidates. (A). Cartoon showing all potential structures in 5' untranslated region. (X: transcripts missing IRES) [Mignone, F., et al., Untranslated regions of mRNAs. Genome Biol, 2002. 3(3): p. REVIEWS0004]. (B) 5' UTRs were identified having IRES sequences in MS candidates from uninfected cells and those challenged with L. pneumophila WT. (C). Ribosome loading of 5' UTRs having IRES sequence in MS candidates from L.pneumophila-infected cells. Displayed are IRES-containing (Red) and IRES-absent (Black) 5'UTRs.

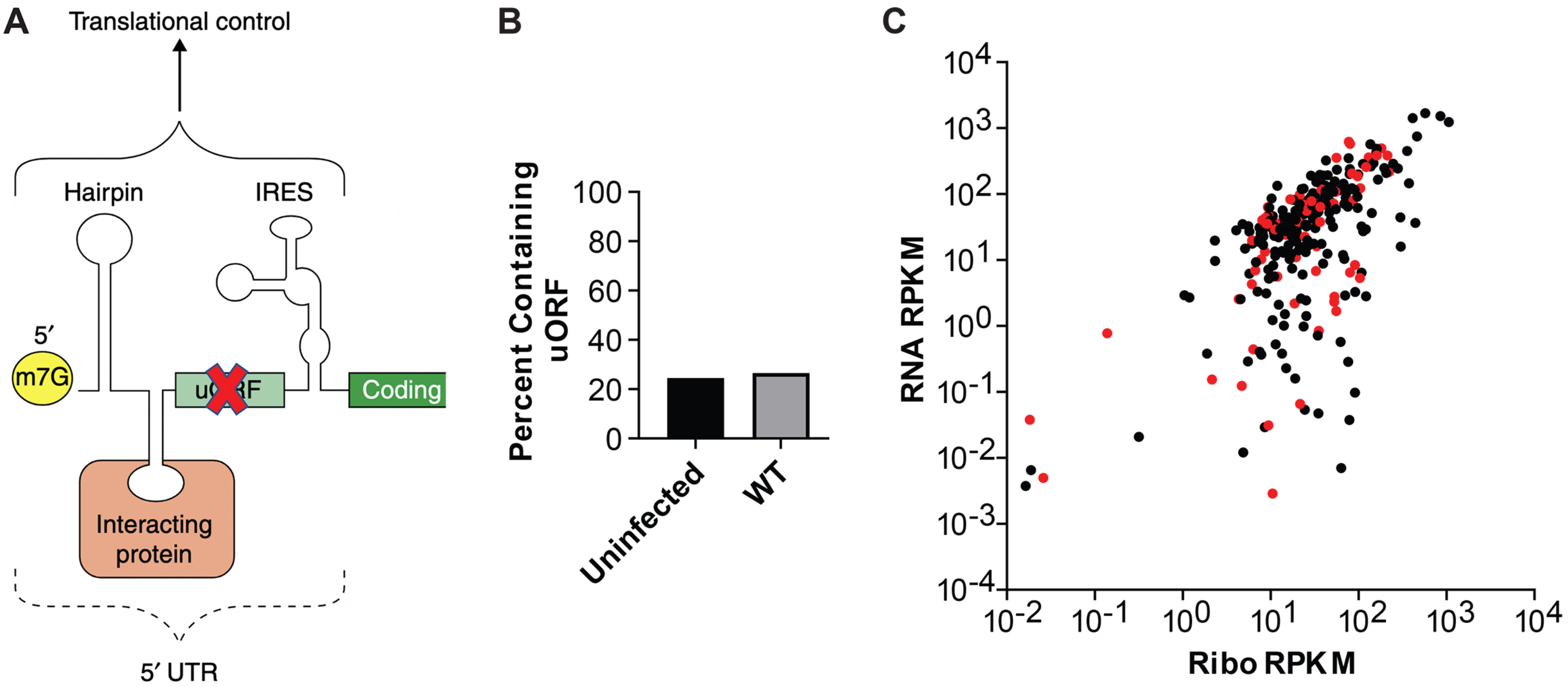

Supplementary Figure 4. uORFs are not enriched in MS candidates. (A). Cartoon showing all potential structures in 5' untranslated region. (X: transcripts missing IRES and or uORFs). [Mignone, F., et al., Untranslated regions of mRNAs. Genome Biol, 2002. 3(3): p. REVIEWS0004] (B). 5' UTRs were identified having uORF sequences in MS candidates from uninfected cells and those challenged with L. pneumophila WT. (C). Ribosome loading of 5' UTRs having uORF sequence in MS candidates from L.pneumophila-infected cells. Displayed are uORF-containing (Red) and IRES-absent (Black) 5'UTRs.
